# Reliability and Validity of a New Survey to Assess Global Health Competencies of Health Professionals

**DOI:** 10.5539/gjhs.v5n1p13

**Published:** 2012-10-22

**Authors:** Mirella Veras, Kevin Pottie, Vivian Welch, Ron Labonte, Javier Eslava-Schmalbach, Cornelia M. Borkhoff, Elizabeth A. Kristjansson, Peter Tugwell

**Affiliations:** 1 Institute of Population Health, University of Ottawa, Ottawa, ON, Canada; 2 Department of Family Medicine, University of Ottawa, Ottawa, ON, Canada; 3 Department of Epidemiology and Community Medicine, University of Ottawa, Ottawa, ON, Canada; 4 Ottawa Hospital Research Institute, Ottawa, ON, Canada; 5 School of Medicine, Universidad Nacional de Colombia, Bogotá, Colombia; 6 Women’s College Research Institute, Women’s College Hospital, Toronto, ON, Canada; 7 School of Psychology, University of Ottawa, Ottawa, ON, Canada; 8 Department of Medicine, University of Ottawa, Ottawa, ON, Canada

**Keywords:** reliability, survey instrument, global health, health inequalities, education

## Abstract

**Objective:**

Health professionals are paying increased attention to issues of global health. However, there are no current competency assessment tools appropriate for evaluating their competency in global health. This study aims to assess the validity and reliability of a global health competency survey for different health disciplines.

**Methods:**

A total of 429 students participated in the Global Health Competency Survey, drawn from family medicine residency, nursing, physiotherapy and occupational therapy programs of five universities in Ontario, Canada. The surveys were evaluated for face and content validity and reliability.

**Results:**

Factor analysis was used to identify the main factors to be included in the reliability analysis. Content validity was supported with one floor effect in the “racial/ethnic disparities” variable (36.1%), and few ceiling effects. Seven of the twenty-two variables performed the best (between 34% and 59.6%). For the overall rating score, no participants had floor or ceiling effects. Five factors were identified which accounted for 95% of the variance. Cronbach’s alpha was >0.8 indicating that the survey items had good internal consistency and represent a homogeneous construct.

**Conclusion:**

The Global Health Competency Survey demonstrated good internal consistency and validity.

## 1. Introduction

Health inequalities between and within countries have increased in recent years (CSDH-Commission on Social Determinants of Health, 2008), due in part to the various impacts of globalization on social determinants of health, including health systems (Globalization Knowledge Network, 2011). Political and economic instabilities, climate change, urbanization, labour market insecurities and shifts in gender roles (despite persisting gender inequalities) are some examples of globalization-related factors that impact health and health systems worldwide (Brewer et al., 2009). Students in Canada and the United States are becoming progressively more interested in global health issues (Hagopian et al., 2008; Redwood-Campbell et al., 2011). This interest has resulted in a proliferation of electives, training and workshops focusing on global health and in programs, institutes and departments in North American and European universities developing global health initiatives (Hagopian et al., 2008). Existing literature on global health focuses largely on the new epidemiological challenges produced by the growth in international trade, travel, and immigration, and little has been written on the question of what global health ought to comprise (Hagopian et al., 2008; Urkin & Henkin, 2001; Nelson et al., 2008; Reed, 2006; Battat et al., 2010). Although there is no consensus on a definition of global health, for this paper we use a broad definition which is expansive enough to incorporate most elements identified by global health scholars: “Global health is an area for study, research, and practice that places a priority on improving health and achieving equity in health for all people worldwide” (Koplan et al., 2009). This definition implies a range of social, political and economic actors, interventions and disciplines; however, our interest lies in those disciplines working within health care settings, and the extent to which they have competencies in global health.

Existing literature suggests that global health competence for health professionals extends beyond clinical skills to incorporate, at a minimum, abilities to work in remote areas and settings with limited resources (Orbinski, 2008; Mill et al., 2010). Present global health training curricula often aim to improve students’ understanding of travel medicine, the global burden of disease, health care disparities, immigrant health, health systems and primary care, as well as teaching them the skills to work with socially disadvantaged populations. Nonetheless, there is no consensus among schools and disciplines on what competencies are adequate for global health (Battat et al., 2010; Evert, 2006; Drain et al., 2007; Fox et al., 2007; Evert et al., 2007; Parsi & List, 2008).

A survey of global health curricula in 17 Canadian medical schools carried out during the, 2005 and, 2006 found that there was a growing demand for global health training, but that the training programs were not responding satisfactorily (Izadnegahdar et al., 2008). Most training programs focused on international electives and only 30% of the schools prepared their students for their overseas practice (Izadnegahdar et al., 2008). At the same time that inadequacies in global health curricula were being documented, the need to expand health professionals’ knowledge of global health was increasing. In 2008, the Canadian Nurses Association, for example, recognized the need to develop nursing leadership in global health and educational programs to support global health education and international exchanges (Tyer-Viola et al., 2009). The World Confederation for Physical Therapy (WCPT) began carrying out several programs and projects for physiotherapists working overseas, as well as supporting international campaigns to endorse the contribution of the profession within global health (World Confederation for Physical Therapy, 2010). Given this interest, how should health professions be prepared in their training for work in the area of global health? Are there unique competency for such work?

To answer these questions and to improve our understanding of global health competencies we surveyed students enrolled in four health disciplines: family physicians, nurses, physiotherapists and occupational therapists. To our knowledge, there was no existing standard questionnaire to measure global health competencies in different disciplines. The instruments identified in the literature measured actual and perceived resident physician knowledge of underserved patient populations in the United States (Wieland et al., 2010), and global health competencies for medical students who participated in overseas electives (Augustincic, 2011). The previous surveys, apart from their focus on one health discipline only, neglected to measure some domains that our review of recent literature on global health identified as potentially important in an assessment of global health competencies. The cross-disciplinary focus of our survey reflects the complex nature of global health itself, with its emphasis on worldwide health issues and the need for interdisciplinary collaborations. This paper describes the development and the assessment of validity and reliability of a global health competencies instrument.

## 2. Methods

Our questionnaire development involved six stages: item selection; a study of the population and setting; survey administration and data collection; analysis of face and content validity; and Exploratory Factor Analysis (EFA) and reliability measurements. We conducted a small pilot test with 36 participants, and then distributed the revised final version of the questionnaire to our full survey population.

### 2.1 Selecting Items

To ensure that all important global health domains were covered, we identified candidate items for the Global Health Competencies (GHC)survey from 4 sources: (a) literature review of instruments used to measure competencies related to global health and health equity for health professionals; b) in-person consultation with six global health and health equity experts; c) on-line consultation with 10 experts in education and global health from different disciplines; d) items from a global health competencies skills survey for medical students (Augustincic, 2011) which used the framework for global health in family medicine (Redwood-Campbell et al., 2011) and the Canadian Medical Education Directives for Specialists (CanMEDS) competency (Frank, 2005); and e) a validated questionnaire used to measure actual and perceived resident physician knowledge of underserved patient populations in the United States that was adapted to the Canadian population (Wieland et al., 2010).

### 2.2 Population and Setting

A total of, 2060 students and residents in five universities within Ontario, Canada were invited to participate in the Global Health competencies online survey. We chose Ontario because it is the country’s most populous and ethnoculturally diverse province, with the highest proportion of immigrants (Townson, 2009) ethnocultural diversity and immigration being defining two aspects of contemporary globalization and, hence, global health. The students were from different disciplines: nursing, physiotherapy, and occupational therapy and family medicine residency programs. The inclusion criteria were: (a) must be a student from the University of Ottawa, University of Toronto, Queen’s University, Western University, or McMaster University; b) must be 18 years or older; c) must be a 1st year student from a master’s program in physiotherapyor occupational therapy, or in the last year of a nursing undergraduate program, or a 1st year resident in a family medicine residency program; d) must provide online informed consent.

### 2.3 Survey Administration and Data Collection

Students were recruited by e-mail through the directors or coordinators of their programs. They received a brief explanation about the study and a web link to access the online survey and consent form. Online surveys have demonstrated superiority over postal surveys in several ways, especially in response speed, response rate and cost efficiency (Sheehan, 2001; VanGeest & Johnson, 2011). Reminders were sent after, 2 and 4 weeks. Data collection periods in each program ranged from one to two months. Data was collected from April, 2011 until October, 2011.

### 2.4 Validity

Validity is defined as the ability of an instrument to measure what it is purported to measure (Swiontkowski et al., 1999) Face validity concerns whether or not the instrument appears to potential test takers to be assessing what it intended to measure (Streiner & Norman, 2005). Content validity reflects the extent to which the measures cover the domains adequately (Streiner & Norman, 2005). We assessed content validity with experts’ opinions, as well as ceiling and floor effects. A floor effect occurs when the respondents provide the lowest possible score for all or almost all items and receive the least desirable score (the proportion of subjects getting the lowest possible score). With a ceiling effect, the opposite occurs: the respondents report the highest score and receive the most desirable score (the proportion of subjects getting the highest possible score) (Stucki et al., 1995). An assessment with good content validity should have few categories with ceiling or floor effects.

### 2.5 Exploratory Factor Analysis (EFA)

Factor analysis was used to identify how many categories were sufficient to gather information contained in the original set of statements, and to explore the interrelationship among the original set of variables (De Villes, 2012). We used exploratory factor analysis to identify the number of common factors influencing a set of measures. Additionally, EFA was used to examine the strength of the relationship between each factor and each observed measure of the GHC survey.

Factor analysis was conducted using principal factor analysis with varimax rotation. First, a sample size of 348 students was included for factor analysis (a ratio of at least five subjects for each one of the variables is recommended). Second, a correlation matrix was developed to determine correlations of r=0.3 or greater. The cut-off point for retained factors used the Kaiser eigenvalue greater than one and Cattell’s scree test rules. In case of disagreement the eigenvalue nearest to 1 was used to define the cut-off point. Finally, within the factors, retained items were selected based on factor loads higher than 0.4 and uniqueness lower than 0.6.

### 2.6 Reliability: Internal Consistency

Internal consistency permits judgment of the reliability of the questionnaire by estimating how well items within a domain fit together (Streiner & Norman, 2005). Internal consistency is based on a single administration of the survey (Streiner & Norman, 2005). Cronbach’s alpha was used to assess the internal consistency of our multi-item instrument. Items with item-total correlation values less than 0.2 were removed. We considered alpha values to be greater than 0.70 as a standard for adequate reliability of the questionnaire (Swiontkowski et al., 1999; Martin et al., 1997).

### 2.7 Data analysis Procedures

Data from the completed surveys were analyzed using the Statistical Package for the Social Sciences (SPSS) software (version 19). Analysis was performed in two steps. Firstly, we described the study participants with descriptive statistics. Secondly, we analyzed the psychometric proprieties–reliability (internal consistency of the survey). Other psychometric proprieties that were evaluated include face and content validity.

## 3. Results

### 3.1 Questionnaire Development and Pretesting

The item selection phase resulted in 86 items relevant to assess global health competencies. Pretesting was conducted to ensure the feasibility of administration. Participants were selected from universities across Canada. In total, thirty six students participated in the pilot testing (5 family physician residents, 6 nursing, 19 physiotherapy, and 6 occupational therapy students).

Reviewers were asked to comment about the content of the questions, difficulty in understanding the questions, response categories for the questions, the logical sequencing of the items, and to report how much time they spent to complete the survey.

### 3.2 Item Reduction and Finalization of Items

Information from the pretesting was used in the process described by Hyland (Hyland et al., 1991) to reduce the number of items, eventually leading to 34 global health competencies items, and 8 demographic items. The items were further reduced after evaluating the reliability of the questionnaire, resulting in 22 global health competencies items, plus 8 demographic questions. Studies have demonstrated that short questionnaires improve response rate (Kellerman & Herold, 2001).

The rationale for excluding items from the survey is discussed in Table 1 (available at: https://docs.google.com/file/d/0BwsAlBgbpA_pQUxrMURCenFRV2c/edit). The final survey was then organized into four parts:

**Table 1:** Item reduction method for global health competency survey

Part of the questionnaire	Original number of questions	Number of excluded questions/number of the questions	Reason to exclude questions	Final number of questions after exclusion
Part 1 -Confidence Level	17	5 (Items: 1.3;1.6;1.4;1.15;1.17)	Problematic wording and missing more than 5% of questions. (This criterion more than 5% is not included in the Hyland criteria).	12
Part, 2- Relevance Level	17	17*	“Poor discriminators removed (items in which 70% or more students endorsed one response- responses were dichotomous. (We only discarded infrequently endorsed items. Discarding frequently endorsed items would have led to nearly all the high impact items being removed.)”. For 5 point scales we excluded questions if they were 70% or more in 2 categories (4 questions included in this case).	0
Part 3 - Global health skills	18	4 (Items: 3.1; 3.4; 3.7;3.10).	Overlapping concepts (in this case we took out complicated wording questions) and responses for items with the same construction and with similar percentage.	14
Part 4- Patient centered attitude	9	9 (4.3=74.2%; 4.6=79.0%; 4.8=75.8%)	Three questions have items in which 70% or more students endorsed one response. Even though the importance of the issue, this section is not directly related to global health education.	0
Part 5- Learning needs about GH	17	9 (Items: 5.1; 5.2;5.4;5.10;5.11; 5.12;5.13; 5.14,5.15)	Overlapping concepts (in this case we took out complicated wording questions) and responses with similar content and percentage; Questions related to didactic methods were excluded because the main propose of the survey is assessing GH competency. Accessing tools could be better addressed in another study after the analysis of the survey and maybe with qualitative methods considering the particularities of each universities resources and students learning preferences.	8
**Total of questions after removing items**				34

* Part 2- Relevance Level – % of poor discrimination question: 2.1 (75%); 2.2(79%); 2.4 (79%); 2.5 (91%); 2.6 (79%); 2.7 (83%); 2.8 (92.5%); 2.9 (79.1%); 2.10 (70.1%); 2.11 (74.6%); 2.13 (74.6%); 2.15 (74.6%); 2.16 (83.6%)

1) Knowledge in global health and health equity;
2) Global health skills for working with patients who have different linguistic, educational, socioeconomic, and cultural backgrounds;
3) Learning needs about global health;
4) Demographic questions. (Survey is available at Table 2 [appendix])

### 3.3 Study Participants

The survey was sent to, 2060 students, of whom 465 agreed to participate (response rate of, 22.6%). Surveys returned without information about the participant’s program of study were excluded from the analysis, leaving a total of 429: 166 family physician residents, and 97 nursing, 68 physiotherapy and 98 occupational therapy students. A survey response rate review demonstrated that survey responses have been decreasing in past decades (Sheehan, 2001). Our study has an average response rate compared with other studies with similar population (VanGeest & Johnson, 2011; Vangeest et al., 2007). Participants for this study were recruited from five universities in Ontario, Canada. The highest response rate was from McMaster University (30.3 %); 82.1% of respondents were female and the majority of the respondents were family physician residents (38.7%). The respondents’ average age was, 26 years (range, 20 to 53years). Most respondents were predominately white (69.2%); were raised by parents who earned $80,001 or more (38.2%), and were fluent in only one language (42.7%) (Table 3).

**Table 3:** Demographics and baseline characteristics of respondents (N=429)

Characteristics	N	%
**Program**		
Family Medicine Residency	166	38.7
Nursing	97	22.6
Physiotherapy	68	15.9
Occupational Therapy	98	22.8
**University**		
University of Ottawa	72	16.8
University of Toronto	88	20.5
McMaster University	130	30.3
Western Ontario University	53	12.4
Queen’s University	86	20.0
**Sex**		
Male	77	17.9
Female	352	82.1
**Age (yrs)**	26.47	
**Background**		
White	297	69.2
Chinese	39	9.1
South Asian	35	8.2
Black	10	2.3
Latin American	4	.9
Southeast Asian	2	.5
West Asian	2	.5
Aboriginal	7	1.6
Other	33	7.7
**Parent’s family income**		
$20,001 to $30,000	21	4.9
$30,001 to $40,000	16	3.7
$40,001 to $50,000	21	4.9
$50,001 to $60,000	35	8.2
$60,001 to $70,000	26	6.1
$70,001 to $80,000	36	8.4
$80,001 or more	164	38.2
Don’t know	110	25.6
**Language able to speak**		
One language	183	42.7
Two languages	171	39.9
Three languages	47	11.0
Four languages or more	28	6.5

### 3.4 Face and Content Validity

Face and content validity of the GHC survey was assessed by convening an expert panel that consisted of 6 global health and health equity experts and 10 experts in education and global health (3 nurses, 1 physiotherapist, 3 occupational therapists and 3 family physicians). The panel assessed the relevance of the included items and suggested additional questions, revisions to the wording of existing questions, and revisions to the sequencing and responses of some items. The intent of this exercise was to eliminate any items that were unclear or too long for participants to complete. After making the recommended changes, we returned the questionnaire for a final approval from the panel of experts.

Although there is no a consensus of cut-off point for ceiling and floor effect (Eechaute et al., 2007), many studies consider floor and ceiling effects will occur when more than one third of the total population has either the best or worst scores, respectively (> 33%) (Eechaute et al., 2007; Barber-Westin et al., 1999). A floor effect was found in one variable (racial/ethnic disparities; 36.1%); seven of the twenty-two variables performed the best (between 34% and 59.6%). For the overall rating score, no participants had a floor or ceiling effect (Table 4).

**Table 4:** Ceiling and floor effect for each domain

Items	Completion rate (%)	% with floor effect *	% with Ceiling effect*
Language barrier	99.8	5.8	34
Income and health	99.8	1.4	59.9
Work and health	99.5	2.6	52.0
SEP and impact on health	100	4.0	50.6
SEP and environmental Health	100	14.2	30.3
Housing and health	99.8	8.4	39.4
SEP and food security	100	13.5	37.5
Racial/ethnic disparities	100	36.1	13.3
Race and clinical decision making	99.5	28	17.5
Gender and access to health care	100	23.1	22.8
Listening	98.6	.9	21.2
Patient background	98.4	1.4	10.5
Discuss sensitive issues	98.6	4	5.6
Identify needs	98.1	2.3	4.4
Health outcome disparities	100	23.8	23.1
Health risks	98.1	0	8.4
Communicable diseases	98.1	0	10.5
Social determinates of health	98.4	0	26.6
Cultural competency	98.8	.5	37.1
Access to clean water	99.1	1.2	29.4
Human rights	99.8	.5	29.4
Global health institutions	99.3	1.9	19.1

* Floor or ceiling effects are present when > 33% of the population marks best or worst score

### 3.5 Factor Analysis

Five factors accounted for 95% variance (Table 5). The scree plot to determine the numbers of factors to be retained is showed in Figure 1. The items with the highest loadings for factor 1 were: “language barrier”, “income and health”, “work and health”, “Socioeconomic position (SEP) and impact on health”, “housing and health”, “SEP and environmental health”, “SEP and food security”, and “health outcome disparities”; for factor, 2: “social determinants of health”, “cultural competency”, “access to clean water”, “human rights” and “global health institutions”; factor 3: “listening”, “patient background”,” discuss sensitive issues” and “identify needs”; factor 4: “racial/ethnic disparities”, “race and clinical decision making”, and “gender and access to health care”; and for factor 5: “health risks” and “communicable diseases” (Table 6).

**Table 5:** Eigenvalues and cumulative variance after principal factor analysis (varimax rotation)

Factor	Variance	Difference	Proportion	Cumulative
Factor1	3.56563	0.36502	0.2566	0.2566
Factor2	3.20061	0.5871	0.2303	0.4868
Factor3	2.61351	0.26497	0.188	0.6749
Factor4	2.34854	1.39961	0.169	0.8439
Factor5	0.94894	0.0401	0.0683	0.9122
Factor6	0.90883	0.41265	0.0654	0.9775
Factor7	0.49618	0.0134	0.0357	10,132
Factor8	0.48278	0.15175	0.0347	10,480
Factor9	0.33103	0.03328	0.0238	10,718
Factor10	0.29775	0.03282	0.0214	10,932
Factor11	0.26493	0.0627	0.0191	11,123
Factor12	0.20223	0.02609	0.0146	11,268
Factor13	0.17614	0.01015	0.0127	11,395
Factor14	0.16599	0.0044	0.0119	11,515
Factor15	0.16159	0.06608	0.0116	11,631
Factor16	0.09551	0.0421	0.0069	11,700
Factor17	0.05341	0.04812	0.0038	11,738
Factor18	0.00529		0.0004	11,742

**Table 6:** Items included in the final version of the global health competencies survey

Factors	Items
Factor 1: Confidence Level in SEP* and Health disparities (8 items)	Language Barrier; Income and Health; Work and health; SEP and impact on health; housing and health; SEP and environmental Health; SEP and food security and Health outcome disparities.
Factor, 2: Social Determinants of Health (5 items)	Social determinants of health; Cultural competency; Access to clean water; Human rights and Global health institutions.
Factor 3: Global Health Skills (4 items)	Listening; Patient background; Discuss sensitive issues and Identify needs.
Factor 4: Health disparities (3 variables)	Racial/ethnic disparities; Race and clinical decision making; Gender and access to health care.
Factor 5: Travel and Migration (2 variables)	Health risks and Communicable diseases.

* Socioeconomic Position

### 3.6 Internal Consistency

Internal consistency of the GHC Survey was calculated using Cronbach’s alpha coefficient, which provides information regarding the strength of inter-item correlation. The reliability analysis of the 22 items obtained a Cronbach’s alpha coefficient of 0.862 (Table 7).

**Table 7:** Internal consistency of the global health competencies survey

Item	Obs	Item-test correlation	Item-rest correlation	Average inter-item correlation	Cronbach’s alpha
Language Barrier	428	0.4613	0.386	0.2245	0.8587
Income and Health	428	0.5632	0.4963	0.2189	0.8548
Work and health	427	0.5225	0.4522	0.2213	0.8565
SEP and impact on health	429	0.5689	0.5027	0.2186	0.8546
SEP and environmental Health	429	0.5973	0.5342	0.2172	0.8535
Housing and Health	428	0.6139	0.5525	0.2163	0.8529
SEP and food security	429	0.6109	0.5494	0.2165	0.853
Health outcome disparities	429	0.5936	0.5305	0.2175	0.8538
Social determinants of health	422	0.5583	0.4915	0.2192	0.855
Cultural Competency	428	0.4503	0.3737	0.2251	0.8592
Access to clean water	425	0.5461	0.4772	0.22	0.8555
Human rights	428	0.5254	0.4548	0.221	0.8563
Global Health Institutions	426	0.4991	0.4262	0.2226	0.8574
Listening	423	0.3097	0.2253	0.2325	0.8642
Patient background	422	0.3368	0.2536	0.2311	0.8633
Discuss sensitive issues	423	0.3561	0.274	0.23	0.8625
Identify needs	421	0.3358	0.2527	0.2312	0.8633
Racial/ethnic disparities	429	0.5581	0.4911	0.2194	0.8552
Race and clinical decision making	427	0.6024	0.5401	0.2169	0.8533
Gender and access to health care	429	0.6107	0.5495	0.2166	0.8531
Health risks	421	0.4625	0.386	0.2244	0.8587
communicable disease	421	0.4676	0.3922	0.224	0.8584
Test	scale			0.2221	0.8626

## 4. Discussion

Face and content validity of the Global Health Competencies Survey were considered adequate according to the panel of experts. The reviewed and approved questionnaire covered the most relevant topics of the global health literature. For content validity, we found one floor effect and few ceiling effects and for overall rating scale we did not find floor or ceiling effects. According to the literature, good content validity is considered when few floor or ceiling effects are found (De Villes, 2012).

The factor analysis confirmed the factor structure of the scale. Five factors were included in the final scale: confidence level in socioeconomic positions and its impact in health outcomes and questions associated with health disparities; social determinants of health; global health skills to work with patients with different backgrounds and characteristics; health disparities; and travel and migration. These global health topics included in the final scale are consistent with our literature review (Battat et al., 2010; Evert et al., 2007). The internal consistency of the GHC Survey was assessed with Cronbach’s alpha. The results indicated that the GHC Survey had good internal consistency (>0.8) for the entire questionnaire including the five factors and all items discriminated well.

The findings of this study showed that the GHC Survey is an appropriate tool for measuring global health competencies in family physician residents, and nursing, physiotherapy and occupational therapy students, and perhaps other health disciplines as well. It is reliable, and it provides a broad range of relevant items capable to measure confidence level in relevant global health issues, global health skills and learning needs in global health.

### 4.1 Implications for Research Use

The GHC Survey has practical utility in research and policy in several ways. The survey can identify knowledge gaps in global health education; contribute to faculties and educators in identifying gaps in their programs; assist in addressing changes in global health education leading to improved training programs; contribute to reduced health inequities by helping to improve global health skills in students’ and residents’ curricula in global health; and address the need for a tools to measure global health competency and open directions for the use and upgrading of this tool. We suggest that educators administer the survey during the first half of a program, as it would help identify progress and highlight unmet learning needs that could potentially be addressed by the program.

### 4.2 Strengths and Limitations

This is the first valid and reliable tool to access global health competencies for different disciplines. The survey demonstrated good internal consistency and validity. The strength of this study is that it contributes to a crucial and emerging literature on global health education by developing and testing a tool to measure global health competencies using a multi-centered and interdisciplinary sample of health professionals from different disciplines. Another advantage of the GHC survey is its administration. It is an online survey, which makes it convenient to administer, and it has a reasonable completion length (about 10 minutes). Our study also had limitations: criterion validation was not assessed, and we administered the survey in English only, despite some health professional training programs in Canada being offered only in French. Despite a low response rate, we did have good representation from a variety of health disciplines.

### 4.3 Implications for Future Research

Future studies are needed to examine the use of this scale to assess effects of programs to increase global health competencies. Confirmatory Factor Analysis (CFA) can be used to confirm the EFA model to evaluate adequately.

Similar analysis of the data obtained in other provinces in Canada would be desirable to assess the scope of the questionnaire performance. The use of the GHC Survey in other countries should be considered with adaptation of the instrument such as language and inclusion of domestic global health issues pertinent to the country. There is also a need for a criterion validation since there is no gold standard questionnaire to assess global health competencies across disciplines.

## Appendix

**Table 2:** Global health competencies survey

Part 1: Knowledge and Interest in Global Health and Health Equity (Self-Assessment)			
For each of the following topics, please indicate how knowledgeable you are in the topic and the level of relevance it has to your education.			

Confidence Level	Not at all confident	Somewhat confident	Very confident
1.1 language barriers and their adverse impact on health and health care.			
1.2 Access to health care for low income nations.			
1.3 The relationship between ethnicity and access to health care in Canada.			
1.4 Health care models that may enhance access to care.			
1.5 The relationship between income and health.			
1.6 The relationship between health literacy (the degree to which individuals can obtain, process, and understand basic health information and services needed to make appropriate health decisions) and health.			
1.7 The relationship between work and health.			
1.8 Mechanisms of how socioeconomic position could impact health.			
1.9 Environmental health and socioeconomic position.			
1.10 Housing and health status.			
1.11 The relation between Food security, socioeconomic position and health.			
1.12 Health outcome discrepancies among different ethnicity, language, religion and cultural beliefs in Canada.			
1.13 Mechanisms for explaining why racial and ethnic disparities exist.			
1.14 Racial stereotyping and clinical decision making.			
1.15 Health equity (a health inequity is an avoidable difference in health between more and less advantaged social groups).			
1.16 Gender and access to health care.			
1.17 Patients characteristics (eg.gender/sex, language, religion, etc.) and access to health care.			

Part 2: Global health skills self-assessment work with patients with different linguistic, educational, socioeconomic, and cultural backgrounds					
Please rate how strongly you agree or disagree with each of the following statements by placing a check mark in the appropriate box.					

	Strongly disagree	Disagree	Neutral	Agree	Strongly agree
2.1 I find it challenging to communicate effectively with my patients with different backgrounds.					
2.2 Listening actively to patients’ concerns were challenging.					
2.3 I am uncomfortable consulting with other health care professionals to address issues of my patients with different backgrounds.					
2.4 Addressing team disagreements related to care for patients with different backgrounds is challenging.					
2.5 It is challenging to provide care to patients with different backgrounds.					
2.6 I am able to understand the perspectives of patients with different backgrounds.					
2.7 It is challenging to discuss sensitive issues (e.g. alcohol, drugs, sexual issues, etc) with my patients with different backgrounds than my own.					
2.8 I find it challenging to identify needs of my patients with different backgrounds.					
2.9 I am aware of the health services available to patients with different Backgrounds.					
2.10 I am effective in completing my clinical responsibilities when working with patients with different backgrounds.					
2.11 Helping patients with different backgrounds to set realistic goals for their health is challenging within the time available.					
2.12 I know how to use the expertise of other health professionals when working with my patients with different backgrounds.					
2.13 I know how to access resources to keep up to date with global health issues.					
2.14 I actively participate in global health activities.					

Part 3:Learners’ Needs about Global Health						
Please rate how important with each of the following statements by placing a check mark in the appropriate box						

	Not at all important	Somewhat important	Neutral	Important	Very important	Extremely important
3.2 Development and implementation of research and scholar activities related to global health.						
3.3 Health risks associated with travel and migration, with emphasis on possible risks and appropriate management, including referrals.						
3.4 Having peer education: student led seminars and journals clubs on global health issues.						
3.5 Knowledge about how travel and trade contribute to the spread of communicable diseases.						
3.6 Relationship between health and social determinants of health, and how social determinants vary across world regions.						
3.7 Cultural competency: understanding how cultural background, socioeconomic status and language barriers can influence access to care and health outcomes.						
3.8 Relationship between access to clean water, sanitation, and nutrition on individual and population health.						
3.9 Understand the relationship between health and human rights.						
3.10 Having a mentor for Global Health training and knowing who are the local global health champions.						
3.11 Having a family physician and/or nurse, occupational therapist and/or physiotherapists supervision on international electives.						
3.12 Have frequent feedback during the learning process related to global health competencies.						
3.13 Have clinical rotations that enable work with disadvantaged or marginalized populations either domestically or internationally.						
3.14 Have a learning guide to help develop and self-evaluate personal global Health competencies.						
3.15 Have training in the social determinants of health and the health issues associated with poverty.						
3.16 Knowledge about how global health institutions (eg. WHO, other United Nations agencies, global institutions) influence health in different world regions through funding and policy.						

Part 4. About you
4.1 In which program are you involved in?
( ) Family medicine residency
( ) Nursing
( ) Physiotherapy
( ) Occupational therapy
4.2 In which university are you taking your program?
( ) University of Ottawa
( ) University of Toronto
( ) McMaster University
( ) Western Ontario University
( ) Queen’s University
4.3. Are you male or female?
( ) Male
( ) Female
4.4 What is your age?
_____ years
4.5 In what country were you born?
( ) Canada
( ) United States
( ) United Kingdom
( ) Germany
( ) Italy
( ) Poland
( ) Portugal
( ) China People’s Republic
( ) Hong Kong
( ) India
( ) Philippines
( ) Vietnam
( ) Other, Please specify:______
4.6 People living in Canada come from many different cultural and racial backgrounds. Are you: (Mark all that apply.)
( ) White
( ) Chinese
( ) South Asian (eg. East Indian, Pakistani, Sri Lankan, etc.)
( ) Black
( ) Filipino
( ) Latin American
( ) Arab
( ) Japanese
( ) Southeast Asian (e.g. Cambodian, Indonesian, Laotian, Vietnamese, etc.)
( ) West Asian (e.g., Afghan, Iranian, etc.)
( ) Korean
( ) Aboriginal Peoples of North America (North American Indian, Métis, Inuit)
( ) Other, please specify: ___________________
4.7 What is the approximate average annual income of your parents/guardians?
( ) $20,000orless
( ) $20,001to$30,000
( ) $30,001to$40,000
( ) $40,001to$50,000
( ) $50,001to$60,000
( ) $60,001to$70,000
( ) $70,001to$80,000
( ) $80,001to$90,000
( ) $90,001to$100,000
( ) $100,001ormore
( ) Don’t know
